# Using Social Media for Social Motives Moderates the Relationship between Post-Traumatic Symptoms during a COVID-19-Related Lockdown and Improvement of Distress after Lockdown

**DOI:** 10.3390/bs13010053

**Published:** 2023-01-06

**Authors:** Giulia Buodo, Tania Moretta, Vieri Giuliano Santucci, Shubao Chen, Marc N. Potenza

**Affiliations:** 1 Department of General Psychology, University of Padova, 35131 Padova, Italy; 2 Institute of Cognitive Science and Technologies (ISTC), National Research Council (CNR), 00185 Rome, Italy; 3 Department of Psychiatry, and National Clinical Research Center for Mental Disorders, The Second Xiangya Hospital of Central South University, Changsha 410011, China; 4 Department of Psychiatry, Yale University School of Medicine, New Haven, CT 06510, USA; 5 Department of Neuroscience, Yale University School of Medicine, New Haven, CT 06510, USA; 6 Department of Child Study Center, Yale University School of Medicine, New Haven, CT 06510, USA; 7 Connecticut Mental Health Center, New Haven, CT 06510, USA; 8 Connecticut Council on Problem Gambling, Wethersfield, CT 06109, USA; 9 Wu Tsai Institute, Yale University, New Haven, CT 06510, USA

**Keywords:** social media, COVID-19, lockdown, social interactions, stress, anxiety, depression, distress, post-traumatic symptoms

## Abstract

Previous research reported inconsistent results on the relationship between social media (SM) use and psychological well-being, suggesting the importance of assessing possible moderators, e.g., motives for SM use. However, no longitudinal study has yet investigated whether, among people who use SM, specific motives for using SM may represent protective/risk factors for the development of psychological distress, especially after a stressful event. Our longitudinal study aimed at assessing the moderating role of motives for using SM (i.e., coping, conformity, enhancement, social motives) in the relationship between COVID-19 pandemic-related post-traumatic stress symptoms during the lockdown and changes in general distress after lockdown. At Time 1 (during the first lockdown in Italy), 660 participants responded to an online survey, reporting their post-traumatic symptoms, motives for using SM, and general distress (i.e., anxiety, depression, and stress symptoms). At Time 2 (three months later, following lockdown), 117 participants volunteered to continue with the follow-up survey assessing general distress symptoms again. Results showed that among those who had experienced more severe post-traumatic symptoms at Time 1, using SM for social motives was associated with more improvement of general distress symptoms. No evidence was found of moderating effects of other motives for SM use. The findings suggest that social connections may have helped to cope with stress during forced confinement, and that SM use may be beneficial for mental health when motivated by maintaining social interactions.

## 1. Introduction

Global reports show that by January 2022, about 5 billion people worldwide were using the internet (i.e., 62.5% of the total world population), with increased interconnectivity influencing virtually every individual and societal behavior. Among people who use the internet, 93.4% use social media (SM; e.g., Instagram, Facebook, YouTube, Twitter), spending on average of 2 h 27 min daily on SM. Adolescents and young adults most actively use SM [1]. Thus, it is not surprising that research has turned its attention to the impact of engaging in SM use on the well-being of individuals. However, the focus of investigation over the last two decades has been mainly on problematic use of SM (PUSM) and its conceptualization and assessment and the exploration of underlying mechanisms (e.g., [2,3,4]).

More recently, an expanding body of research has pointed to the association between SM use and social, emotional, and personal well-being (see [5,6,7]). However, both cross-sectional and longitudinal studies initially provided mixed findings, with some suggesting negative associations between SM use and well-being, and others showing positive links or highlighting the moderating roles of some individual differences ([8,9]). Recent meta-analytic work has provided a clearer understanding, suggesting that SM use is linked to small but significant declines in well-being (see [10,11]). However, this conclusion has been challenged with some suggestions that roles of psychological processes that may drive people’s SM use had been neglected or marginalized in most studies. In other words, SM use may be neither “good” nor “bad” in itself, and rather, SM use may enhance or diminish well-being depending largely on the patterns and motives for their use ([12,13,14]). For instance, it has been reported that using SM passively (“scrolling”, i.e., monitoring others’ SM profiles with little or no direct communication with them) or for procrastination or escape may be related to more negative measures (e.g., loneliness), while active use and social motives (i.e., communicating and interacting with others by posting comments and sharing information) may be related to more positive measures (e.g., more perceived social support, higher self-esteem, and greater life satisfaction [15,16]). Interestingly, and fitting with these findings, a deeper look into the links between PUSM and specific motives for using SM (coping, i.e., to reduce negative feelings; conformity, i.e., in response to peer pressure; enhancement, i.e., to increase positive affect; and social, i.e., to improve contact and relationships with friends) revealed that while coping and conformity are strong predictors of PUSM, social motives are not, thus strengthening the idea that using SM for connecting to other people and maintaining or increasing meaningful social relationships may be in itself beneficial rather than detrimental to the individual’s well-being ([17,18,19]).

With the outbreak of the COVID-19 pandemic in early 2020, countermeasures were taken to contain viral spread. These measures included spatial distancing, travel bans, stay-at-home orders, and lockdowns. As a consequence of these restrictions, a dramatic and sudden change in the lifestyles of billions of people occurred, affecting work, education, leisure, and, crucially, requiring the suspending or severe limitation of in-person social interactions. As such, the average daily time spent on SM by adults and youth in the US and Europe has shown increases since the start of the COVID-19 pandemic, and usage has remained consistently increased to the present day as compared with 2019 and earlier ([20,21]).

Besides concerns regarding the pandemic’s threats to people’s physical health and economic stability, the psychological impact of traumatic experiences, both in shorter and longer terms, has garnered attention by researchers worldwide. In particular, physical confinement at home under pandemic control measures has been associated with loneliness, anxiety, depressive and post-traumatic symptoms among children, adolescents, general adults, and older adults (e.g., [22,23,24,25,26,27]). Over time, though, people have largely demonstrated resiliency and abilities to effectively manage psychological challenges related to the pandemic, notwithstanding important interindividual differences [28]. Considering that social connectedness may have a buffering effect against the negative outcomes of stressful events, including a pandemic [29], and considering that using SM for social motives may boost subjective well-being, some have explored whether using SM for social vs. other motives during the COVID-19 pandemic has been related to better measures of psychological well-being. Indeed, the available findings suggest that specific motives for using SM may have different effects. For example, social connection motives (including bonding with similar-minded people and creating and trusting online peer support networks) and active use were positively related to well-being, whereas information-seeking and passive use were associated with poorer well-being [30,31,32]. However, a comprehensive evaluation of the role of different motives for SM use, drawing from a theory-driven model and taking motivation and affect into account, has not yet been undertaken in the context of SM use during the pandemic. A theoretically based understanding of the motives (i.e., the psychological needs) underlying online activities can provide a strong conceptual background for assessing the unique contribution of specific motives for SM use (see [17,18]). In the present study, we referred to the motivational model of internet use [17,18,19] that considers four basic motives: coping, conformity, enhancement, and social.

The present longitudinal study was aimed at assessing whether and how specific motives for using SM (coping, conformity, enhancement, and social) during the lockdown moderated the relationship between COVID-19-pandemic-related post-traumatic symptoms and changes in general distress symptoms (specifically, anxiety, depression, and stress symptoms) measured post- (vs. during) lockdown. In particular, we sought to elucidate whether people experiencing COVID-19-pandemic-related traumatic stress who used SM for social motives during forced confinement experienced a reduction of general distress symptoms over time. To investigate, we used an online survey for collecting self-reported pandemic-related post-traumatic stress symptoms, motives for using SM, and general distress symptoms during the first lockdown period in Italy (Time 1). Three months later, during the lockdown release (Time 2), general distress symptoms were assessed again.

Drawing on the existing literature, we hypothesized that the relationship between post-traumatic symptoms related to the COVID-19 pandemic during the lockdown and changes in general distress symptoms after the lockdown would be moderated by the motives for using SM during the lockdown. Specifically, using SM was expected to be associated with a reduction of general distress symptoms over time when driven by social motives, due to the beneficial effects of strengthened social connectedness in a situation in which face-to-face social interactions were either prohibited or restricted. Conversely, using SM primarily for coping, conformity or enhancement was expected to be associated with worsening of general distress symptoms over time.

## 2. Materials and Methods

### 2.1. Participants

The present longitudinal study was conducted in Italy. Data were collected in two phases (i.e., during the first COVID-19 lockdown phase [Time 1; from April to early May 2020] and following lockdown release [Time 2; July–August 2020]). The study is part of a larger longitudinal project on the relationship between psychological distress during the COVID-19 pandemic and PUSM, from which another paper was published with different objectives and methods [33].

Participants were recruited by advertising the survey on online research recruitment sites and through snowball sampling using social media (i.e., the survey was posted on social media, and readers were encouraged to forward the research opportunity to other potential participants). Those who agreed to participate were required to read an informed consent form and provide consent before continuing. After the informed consent, the survey started with a section on demographic information including questions about participants’ sex, age, education, and monthly income. Only respondents older than 18 years were given the possibility to continue to the online survey. Additionally, respondents were required to indicate their demographic and COVID-related information, and to provide their e-mail address to be contacted again for taking part in a follow-up survey. The section on demographic data was followed by self-report measures of COVID-19-pandemic-related post-traumatic stress symptoms, motives for using SM, and general psychological distress. At Time 2, general psychological distress was assessed again.

The survey was set in such a way that participants had to answer all questions before they were allowed to submit their answers, but they could quit the survey at any time. No participant quit before the end of the survey at both Times 1 and 2.

At Time 1, 660 people completed the survey. Of these, 303 provided their e-mail address agreeing to be re-contacted for the second phase of the study. At Time 2, they were e-mailed by the second author and invited to take part in the second phase. At Time 2, 117 respondents proceeded to take the survey a second time. Therefore, a sample of 117 participants took part in the study at both Times 1 and 2. Given that we were interested in changes from Time 1 to Time 2, we only describe data from these 117 participants. The demographic characteristics of the sample are presented in Table 1.

### 2.2. Self-Report Measures

To assess general distress symptoms, participants were asked to complete the Depression Anxiety Stress Scales-21 (DASS-21; [34,35]). The DASS-21 assesses general distress through three separate subscales (i.e., anxiety, depression, and stress). It includes 21 items rated on a 4-point scale (0 = Did not apply to me at all; 1 = Applied to me to some degree, or some of the time; 2 = Applied to me to a considerable degree or a good part of time; 3 = Applied to me very much or most of the time). Scores are considered as clinically significant when equal to or over 5 for the depression subscale, equal to or over 4 for the anxiety subscale, and equal to or over 8 for the stress scale [36]. The Italian version of the DASS-21 has been reported to be a robust measure of anxiety, depression, and stress [35]. In our sample, the DASS-21 Cronbach’s alpha was excellent (α = 0.95).

To assess COVID-19-pandemic-related post-traumatic stress symptoms, participants were asked to complete the Impact of Event Scale-Revised (IES-R; [37,38]). The IES-R is a self-report measure of subjective distress for different specific life events in the past seven days. It includes 22 items that are based on the diagnostic criteria for Post-Traumatic Stress Disorder (PTSD) reported in the fourth edition of the Diagnostic and Statistical Manual of Mental Disorders. A score between 24–36 reflects mild-to-moderate post-traumatic stress disorder (PTSD) and a score equal to 37 or above reflects severe PTSD ([39]). The IES-R has been found to be a reliable and valid instrument to measure traumatic stress symptoms in the context of viral outbreaks (e.g., COVID-19; see [39,40]). In our sample, the IES-R Cronbach’s alpha was excellent (α = 0.90).

To assess motives for using SM, participants were asked to complete the Social Media Motives Questionnaire (SMMQ), a version of the Facebook Motives Questionnaire [17] adapted to a more general SM context, i.e., by replacing the word “Facebook” with the words “social media”. Specifically, participants were asked how often they logged on one or more SM for different motives, thinking of all the times they had been using SM during the past week (12 months in the original version). The scale includes four motives: coping (e.g., “To forget about your problems?”), conformity (e.g., “To not feel excluded?”), enhancement (e.g., “Simply because it is fun?”), and social (e.g., “To share a special occasion with friends?”). The questionnaire includes 16 items rated on a 5-point scale. Higher scores indicate higher levels on each motive. In our sample, the Cronbach’s alphas for the total score and for each motive subscore were acceptable (Total: α = 0.89; Coping: α = 0.89; Conformity: α = 0.67; Enhancement: α = 0.75; Social: α = 0.87).

#### Statistical Analysis

All analyses were performed using R software [41]. Pearson correlations were calculated between study variables (i.e., COVID-19-pandemic-related post-traumatic stress symptoms at Time 1, as indicated by IES-R scores; general distress symptoms improvement, as assessed by computing the DASS-21 total scores at Time 1 minus total scores at Time 2; motives for using SM, as indicated by the coping, conformity, enhancement, and social motives scores on the SMMQ). The contributions of COVID-19-pandemic-related post-traumatic stress symptoms, specific motives of using social media, and their two-way interactions (i.e., predictors at Time 1) to general distress symptom improvement (i.e., the dependent variable) was assessed by the Bayesian approach, which is considered a powerful procedure for testing hypotheses in psychological research [42,43]. In fact, Bayesian parameter estimation is not affected by the sampling plan [44]. In the present study, Bayesian adaptive sampling for variable selection and model averaging was used to assess what combination of statistical predictors provided an adequate description of the distributions that generated the observed change in general distress symptoms (Time 1). As an extension of Bayesian inference, this approach considers parameter uncertainty through prior distribution and model uncertainty and obtains posterior distributions for the model parameters and the model by using Bayes’ theorem, allowing for model selection and combined estimation [45,46]. Specifically, 512 models (taking sex and age into account) were estimated by a Markov chain Monte Carlo sampling method by using the Zellner–Siow Cauchy prior on the coefficients, and a uniform prior distribution over the models. Multicollinearity was monitored by examining the variance inflation factor (VIF). The null hypothesis was rejected when the 95% Bayesian credibility intervals (BCIs) did not include the null value [47].

## 3. Results

As shown in Table 2, Pearson’s correlation coefficients showed that improvement of distress symptoms after lockdown was associated with higher COVID-19-pandemic-related post-traumatic stress symptoms and greater use of SM for coping, conformity, enhancement, and social motives. COVID-19-pandemic-related post-traumatic stress symptoms and using SM for coping, conformity, enhancement, and social motives were positively associated with each other. The VIF indicated that multicollinearity was not a concern (IES-R total score: VIF = 1.27; coping: VIF = 1.80; conformity: VIF = 1.56; enhancement: VIF = 1.68; social motive: VIF = 1.32).

The hypothesis regarding a possible moderating effect of motives for using SM on the relationship between COVID-19-pandemic-related post-traumatic symptoms during the lockdown and changes in self-reported general distress symptoms after the lockdown was tested, and only the interaction social motive × COVID-19-pandemic-related post-traumatic stress symptoms had marginal pip > 0.5 (see Table 3).

**Figure 1:**
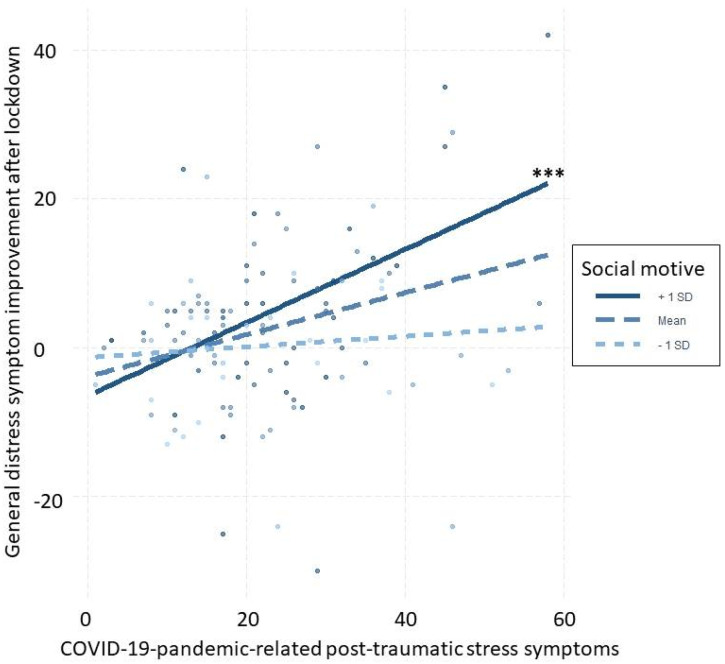
Moderation of using SM for social motives on the relationship between improvement of general distress symptoms after lockdown and COVID-19-pandemic-related post-traumatic stress symptoms. *** statistically significantly different slopes (*p* value < 0.001).

Using SM for social motives moderated the relationship between improvement of general distress symptoms after lockdown and COVID-19-pandemic-related post-traumatic stress symptoms (β = 0.03, 95% BCI = [0.02; 0.04]). Specifically, for those who had high levels of COVID-19-pandemic-related post-traumatic stress symptoms, using SM for interacting with other people during the lockdown was associated with more improvement of general distress symptoms after the lockdown (Figure 1). There was no evidence in favor of other statistical predictors.

## 4. Discussion

The COVID-19 pandemic has had devastating effects on the physical and mental health of people the world over. A full understanding of such effects remains incomplete and, considering that the global public health crisis is far from over, it is of considerable importance to elucidate which factors have the potential to improve or to worsen psychological health. The use of SM has increased during social confinement measures that were taken to limit viral spread. While the numbers do not provide by themselves explanations for changes in use of SM, pre-pandemic research on both the motives for using SM, e.g., [17,18] and the role of different patterns and motives of use on the link between SM use and individuals’ well-being [15,16] may offer a background for hypothesis generation and testing. Specifically, we hypothesized that using SM for social motives during the lockdown would moderate the relationship between post-traumatic symptoms experienced during lockdown and improvement in general distress symptoms post- vs. during the lockdown. Conversely, using SM for coping, conformity, or enhancement was expected to be associated with worsening of general distress symptoms over time.

In line with our hypothesis, the findings show that using SM to improve contact and relationships with friends moderated the relationship between COVID-19-related post-traumatic symptoms during lockdown and general distress symptoms after lockdown. Specifically, using SM for social motives statistically predicted the reduction in general distress symptoms among those who reported stronger COVID-19-related post-traumatic symptoms. Of note, if only the mean IES-R value is considered in our sample (i.e., 22.53), we should conclude that COVID-19-related post-traumatic symptoms were, on average, within the normal range. However, what is to be noted in our findings is that using SM for social motives statistically predicted the reduction in general distress symptoms among those who reported more COVID-19-related post-traumatic symptomatology. Thus, for those who may have experienced stronger psychological impacts of the COVID-19 pandemic, using SM specifically for maintaining and/or expanding social contacts seemingly represented an effective way to relieve anxiety, depression, and stress symptoms over time. Importantly, the other motives (i.e., decreasing negative mood, enhancing positive mood, and conformity) did not demonstrate significant contributions in moderation models. This finding lends support to the idea that the use of SM seems to be specifically associated with the motivation to pursue social connectedness, unlike PUSM, that seems instead to be more strongly driven by motivations related with diminishing negative affect (coping)/enhancing positive affect (enhancement) [14,18,48]. Furthermore, and perhaps most importantly, our results not only extend previous evidence suggesting that during lockdown, SM use has provided a way for individuals to stay connected despite spatial distancing, but also shows that the positive effect on well-being of using SM for social motives seems not to be the same for all individuals impacted by the COVID-19 pandemic, but specifically for those who reported more post-traumatic symptoms. It may be that the possibility offered by SM to compensate (at least partially) for the lack of offline social interactions allowed people with more severe symptoms to cope effectively with social disconnection and such negative emotions as anxiety, loneliness, and fear, that may constitute risk factors for developing mental health problems, and PTSD in particular [49]. Considering that social integration and social support may buffer against adverse psychological effects of large-scale disasters, including the COVID-19 pandemic [50], our findings suggest that social connections, not SM use in and by itself, may be key to how people cope with stress in times of crisis, and that SM use may have been beneficial by enabling maintenance of social contacts in such a collective stressful condition as forced social confinement. In a broader perspective, our and others’ research on SM use and well-being during the COVID-19 pandemic contributes to a shift toward a more nuanced understanding of the potential benefits and risks of SM use. The dichotomy of whether SM can help or harm psychosocial health may be overly simplistic, and considering the who, why, and how of SM use appears relevant [51]. Instead of viewing SM use as either “good” or “bad” for an individual’s well-being, longitudinal studies and finer-grained assessments of contextual and individual factors, including motives for SM use, are warranted to ultimately improve our understanding of the impacts of SM on people’s mental health.

Overall, we believe that the significance of the present study lies in the fact that it provided a novel contribution to understanding the relationship between SM use and well-being, with particular reference to the potential psychological impact of the COVID-19 pandemic, by highlighting that social motives were uniquely associated with the reduction of general distress symptoms. Additionally, the use of a theory-driven framework and a Bayesian approach for data analysis represent considerable strengths of the study.

The results of the present study should be interpreted in light of some limitations. First, the sample was not homogeneous with respect to gender distribution. Although the present study is the first to our knowledge to assess the moderating effects of motives for SM use on changes in psychological distress during vs. post-lockdown, future studies with larger, gender-balanced samples are warranted to further explore this issue, including possible gender-related differences. Second, data were collected through online surveying, in part due to the COVID-19-pandemic-related restrictions. Indeed, in-person data collection (e.g., structured interviews) in controlled research settings has several methodological advantages over its online counterpart. However, online data collection provided a reasonable compromise between feasibility and validity during the COVID-19 pandemic.

The psychological toll of the COVID-19 pandemic has been particularly high among youth, the largest social group that uses SM [52]. It is important that researchers and policymakers intensify efforts for understanding the long-term effects of the pandemic on youth’s mental health, the complex set of unprecedented stressors that can challenge psychological and social well-being, the mechanisms through which those stressors operate, and the factors that may serve as buffers from triggering or worsening psychological distress throughout a prolonged COVID-19 pandemic [53]. In this regard, effective individual coping strategies as well as collective initiatives of self-organization, both offline and online, should receive support to help mitigate potential psychosocial impacts of the pandemic on the “digital native” generation and others [54].

## 5. Conclusions

It is important to expand our understanding of how motives for SM use may influence the impact of SM use on individuals’ well-being. In particular, our longitudinal study shows that using SM for social motives moderates the relationship between stronger COVID-19-pandemic-realated post-traumatic symptoms and decrease in general distress symptoms over time. Other motives did not moderate this relationship. It seems likely that people used SM as a way to keep pursuing social interactions that had to be discontinued during the lockdown. This strongly suggests that it is not SM use in itself, but rather engaging in social interactions, that may help mitigating adverse effects of stress. This is a key issue deserving due attention in a constantly evolving socio-technological world that often neglects or underestimates the beneficial role of social relationships for emotional well-being and recovery from stressful life events.

## Figures and Tables

**Table 1:** Demographic characteristics of the sample participating at both Times 1 and 2.

		N (%)/Mean (SD)
		Sample N = 117
Gender	Female	N = 91 (78%)
	Male	N = 26 (22%)
	Age, Years	31.41 (11.00; range = 18–72)
Education	Junior high school	N = 1 (1%)
	Senior high school	N = 40 (34%)
	Bachelor’s	N = 31 (27%)
	Master’s Degree	N = 32 (27%)
	Specialization	N = 2 (2%)
	PhD	N = 11 (9%)
SES	High	N = 2 (2%)
	Mean High	N = 18 (15%)
	Mean Low	N = 33 (28%)
	Very Low	N = 27 (23%)
	Student	N = 37 (32%)
Social media mainly used	WhatsApp	N = 94 (80%)
	Facebook	N = 89 (76%)
	Instagram	N = 78 (67%)
	Skype	N = 23 (20%)
	Messenger	N = 19 (16%)
	Telegram	N = 11 (9%)
	TikTok	N = 5 (4%)
	Twitter	N = 7 (6%)
	FaceTime	N = 7 (6%)
	Tinder	N = 2 (2%)
	Snapchat	N =2 (1.7%)
	IES-R	22.53 (11.99)
SMMQ	Coping	4.44 (4.01)
	Conformity	2.15 (2.66)
	Enhancement	4.62 (3.20)
	Social Motive	8.16 (4.69)
	DASS-21 Time 1	17.11 (13.28)
	DASS-21 Time 2	14.62 (13.87)

List of abbreviations: SES = Socioeconomic Status; IES-R = Impact of Event Scale-Revised; SMMQ = Social Media Motives Questionnaire; DASS-21 = Depression Anxiety Stress Scales-21.

**Table 2:** Intercorrelations between the study variables.

Subsample That Participated at Both Time 1 and 2, N = 117						
	1.	2.	3.	4.	5.	6.
1. Distress symptom improvement	1					
2. IES-R	0.28 **	1				
3. Coping	0.12	0.44 ***	1			
4. Conformity	0.13	0.25 **	0.44 ***	1		
5. Enhancement	0.01	0.17	0.55 ***	0.48 ***	1	
6. Social motive	0.19 *	0.06	0.27 **	0.45 ***	0.38 ***	1

*** = *p* value < 0.001; ** = *p* value < 0.01; * = *p* value < 0.05.

**Table 3:** Bayesian analyses for assessing the moderating effects of motives for using social media on the relationships between improvement of general distress symptoms after lockdown (the dependent variable) and COVID-19-pandemic-related post-traumatic stress symptoms.

N = 117	Model I		Models		
	pip *	Post β **	I	II	III
IES-R ***	0.17	0	0	0	0
Coping	0.16	0	0	0	0
Conformity	0.18	0	0	0	0
Enhancement	0.27	0	0	0	1
Social motive	0.31	0	0	1	0
IES-R × Coping	0.15	0	0	0	0
IES-R × Conformity	0.16	0	0	0	0
IES-R × Enhancement	0.18	0	0	0	0
**IES-R × Social motive**	**0.97**	**0.03**	**1**	**1**	**1**
Bayesian Factor			1	0.42	0.41
R^2^			0.14	0.16	0.16
Posterior probabilities			0.18	0.08	0.07

Subsample that participated at both Time 1 and 2; the dependent variable = distress symptom improvement after lockdown (DASS-21 score at Time 1 minus DASS-21 score at Time 2). * “Pip” stands for marginal posterior inclusion probabilities. ** “Post β” stands for the posterior coefficient. For each model, the included predictors are indicated as either “1” or “0”, where “1” represents inclusion of the predictor in the model and “0” represents its exclusion. *** IES-R stands for the Impact of Event Scale-Revised total score. Coping, Conformity, Enhancement, and Social Motive are the four subscales reflecting the main motives for using social media included in the Social Media Motives Questionnaire. Note that the moderating effect of using social media for social motives appeared as a predictor of distress symptom improvement after the lockdown in all three top models (i.e., the models with the highest Bayesian factors).

## Data Availability

The data presented in this study are available on reasonable request from the corresponding author. The data are not publicly available due to ethical concerns.
